# Editorial: Images from red cell

**DOI:** 10.3389/fphys.2022.1113951

**Published:** 2023-01-12

**Authors:** Paola Bianchi, Giampaolo Minetti, Anna Bogdanova, Lars Kaestner

**Affiliations:** ^1^ Hematology Unit, Physiopathology of Anemias Unit, Fondazione IRCCS Ca’ Granda Ospedale Maggiore Policlinico Milano, Milan, Italy; ^2^ Red Blood Cell Research Group, Institute of Veterinary Physiology, and Center for Clinical Studies (ZKS), Vetsuisse Faculty, University of Zurich, Zurich, Switzerland; ^3^ Zurich Center for Integrative Human Physiology (ZIHP), Zurich, Switzerland; ^4^ Department of Biology and Biotechnology “L. Spallanzani”, Laboratories of Biochemistry, University of Pavia, Pavia, Italy; ^5^ Theoretical Medicine and Biosciences, Medical Faculty, Saarland University, Homburg, Germany; ^6^ Dynamics of Fluids, Experimental Physics, Saarland University, Saarbruecken, Germany

**Keywords:** red blood cell morphology, anemia, membrane-skeleton, ion channels, reticulocyte, membrane vesicles

Red blood cell (RBC) morphology is always a fascinating Research Topic. Being the most abundant cells in the organism that are easily collected, RBCs have been the object of research since the beginning of cellular morphological investigations (Bessis, 1974; Bain, 2005; Invernizzi et al., 2020). The many different shapes of RBCs reflect not only the cell integrity/abnormality but also its age and physical state. The different shapes of RBCs inspired morphologists, pathologists, and hematologists in naming different pathogenic conditions. Because of their “apparent” simplicity, resulting from the loss of all intracellular organelles that is associated to physiological deformability, fundamental for their squeezing through narrow capillaries of the microvasculature and splenic slits, RBCs have been considered for many years as mere hemoglobin-containing bags fit for oxygen delivery to and CO_2_ removal from peripheral tissues.

However, new advancements in the research on cell ion balance, membrane transporters and cytoskeletal structure, and in studying RBC morphology, as well as novel insight into erythroid maturation, have shed new light on more detailed features of the physiology of these cells, reviving the interest in this cell that has served for decades as a model for studies of cell biology, membrane architecture and dynamics, pathophysiology, and drug delivery (Kaestner 2004; Kaestner and Minetti, 2017).

In the Research Topic—“*Images from Red cells*”, we provided a platform for exposing the main advancements in the ongoing RBC research. Twelve papers were published in the first volume. Inspired by this success, the second volume under the same title was initiated.

The opening article (Simionato et al.) focused on traditional and recent approaches to the study of RBC morphology. The new approaches, including 3D evaluation combined with artificial intelligence, ektacytometry and next-generation sequencing, suggest that not always the morphological-based nomenclature of some defects is sufficiently accurate and exhaustive in describing the pathogenic mechanisms involved. This is the case, for example, for band 3 protein defects that may result in spherocytosis, ovalocytosis or stomatocytosis, depending on the position of causative mutations (Bruce, 2006; Flatt and Bruce, 2018).

A very extensive overview of RBC morphology in different species is given by Benga and Cox, who evaluated RBCs by light- and scanning electron-microscopy. They focused on the water channel protein, aquaporin1, that ensures the exchange of water across the RBC membrane, as required in different species in relation to their physical activity, metabolic rate, and their mean blood flow rate.

Imaging RBCs is commonly performed on RBCs that are fixed and placed on a coverslip, thus precluding their dynamic imaging in action, e.g., *in vivo* (Kihm et al., 2021) or during measurements of their sedimentation rate *in vitro*. The latter is of particular interest since the model involving the sedimentation of RBC-aggregates was replaced by a percolating gel-based colloidal physics theory (Darras et al., 2022a). In their new contribution, Darras et al. present different imaging approaches, providing visual evidence for the breakdown of the percolating gel formed by RBCs as the mechanism underlying sedimentation during ESR measurements.

In recent years, ion channels represent an intriguing Research Topic of research associated with RBC morphology and cell volume regulation (von Lindern et al., 2022). Piezo1, for example, is a non-specific cation channel involved in RBC volume regulation; gain-of-function mutations in its gene are responsible for hereditary xerocytosis. Recent results based on high-resolution atomic force- and confocal-microscopy studies, indicate that this protein is distributed in the RBC membrane in a non-uniform manner (Dumitru et al., 2021). Božič and Svetina developed a mathematical model by minimizing the bending energy and the free energy of freely-moving membrane inclusions and used it to confirm the non-homogeneous distribution of Piezo1 molecules in the RBC membrane.

Aberrant maturation of RBCs from reticulocytes is involved in some RBC pathologies (Moura et al., 2020). The morphological changes during this process have been the object of different studies. During maturation, reticulocytes reduce their plasma membrane, removing or degrading residual internal organelles, proteins and membranes. By comparing the profile of pure cultured reticulocyte preparations and cells from different diseases, Stevens-Hernandez et al. showed that the size and composition of the different cells correlate with the different stages of reticulocyte maturation. A tight relationship between RBC volume, amount of intracellular unremoved proteins and immaturity has been observed in overhydrated hereditary stomatocytosis. Using transmission electron microscopy, Dussouchaud et al. followed ultrastructural changes of RBC precursors (erythroblasts) upon *ex vivo* human erythropoiesis. They showed that mitochondria are progressively cleared between the polychromatophilic erythroblast (Poly-E) to orthochromatophilic erythroblast (Ortho-E) stages. Furthermore, the intracellular vesicle trafficking depicts changes in endosomes and exosomes during the basophilic erythroblast to Poly-E transition; autophagosomes, in particular, are increased from pro-erythroblast to Ortho-E stages. The combined use of fluorescence labeling and micro-manipulation of RBCs has proven to be a powerful tool for studying the development of the bilayer-associated membrane skeleton in primary embryonic erythroid cells. Using this approach, Delgadillo et al., demonstrated that the localization of membrane-skeletal components of mammalian erythroid cells during their development is insufficient by itself to produce a mature membrane-skeleton, and that additional, subsequent, processes are required to strengthen intra-skeletal interactions.

Extracellular vesicles have been in the focus of other contributions in this Research Topic. They are cell-derived membrane particles including exosomes, ectosomes, microvesicles and microparticles. Some of them are formed during apoptosis. Vesicles are often involved in intercellular communication or in the transport of macromolecules between cells. Nguyen et al. tested the ability of RBC-derived vesicles to serve as potential drug carriers by loading them with DNA plasmids coding for the green fluorescent protein. The modified vesicles were used to transfect THP-1-derived macrophages, which were then analyzed by fluorescence microscopy and flow cytometry. Although the treated vesicles were almost completely taken up by macrophages, the expression of the green fluorescent protein was only observed in a subpopulation of macrophages.

Usually, membrane vesicles are generated *in vitro* in media of extremely low tonicity. However, to maintain the concentration of substrates for various transporters and enzymes within the physiological range, vesicles should be suspended in media of higher tonicity. Tiffert and Lew investigated, by electron microscopy, the effects of these hypertonic changes on vesicle morphology. They demonstrated that hypertonic transitions cause an irreversible osmotic collapse of sealed membrane vesicles. Awareness of these phenomena is critical for the interpretation of experimental results.

Microenvironmental conditions associated with RBC storage, the presence or absence of the spleen, as well as the changes in the intraerthytrocytic master regulators such as the redox balance and energy status (ATP concentration), have impact on RBC morphology. Cold storage of RBCs is associated with the accumulation of various morphological and functional alterations. Small “micro-erythrocytes” that accumulate during storage in variable amounts from donor to donor are cleared rapidly after transfusion and their proportion correlates with transfusion recovery. Investigating these changes is of utmost importance in transfusion medicine. Marin et al. studied and quantified micro-spherocyte formation by imaging flow cytometry. These strategies would further facilitate physiologically-relevant quality control of erythrocyte concentrates.

Sickle cell disease, caused by a point mutation in the beta-globin gene, got its name from a typical RBC morphology associated with the formation of aggregates of sickle hemoglobin. In a cooperative study, Peretz et al. investigated in these patients the association between the spleen status (hyper/hypo or asplenism) with clinical, laboratory and morphological parameters.

Finally, attention has been given to the redox properties of human RBCs, and in particular to their adaptation to vitamin C (ascorbic acid) recycling. In their study, Eigenschink et al. showed that the uptake of dehydroascorbate, the fully oxidized form of this vitamin, largely affects the redox metabolism of human RBCs by lowering cellular levels of reactive oxygen species and elevating the plasma membrane electron transport activity.

All the above-mentioned contributions to the second volume of “*Images from Red Cell*” provide evidence to the general notion that “seeing is believing”. We trust that this new article Research Topic will set a milestone and prove that the story continues.
